# Supplementary material for: Warsaw Breakage Syndrome associated DDX11 helicase resolves G-quadruplex structures to support sister chromatid cohesion
Source: Nat Commun. 2020 Aug 27;11:4287. doi: 10.1038/s41467-020-18066-8 (PMC7452896; doi:10.1038/s41467-020-18066-8)
Supplement: Supplementary file 1 — Supplementary Information [file 41467_2020_18066_MOESM1_ESM.pdf]

Warsaw Breakage Syndrome Associated DDX11 Helicase Resolves G-Quadruplex Structures to Support Sister Chromatid Cohesion

Van Schie / Faramarz et al., *Nature Communications* 2020

Supplementary Figures 1-13

Supplementary Table 1

1

Supplementary Figure 1

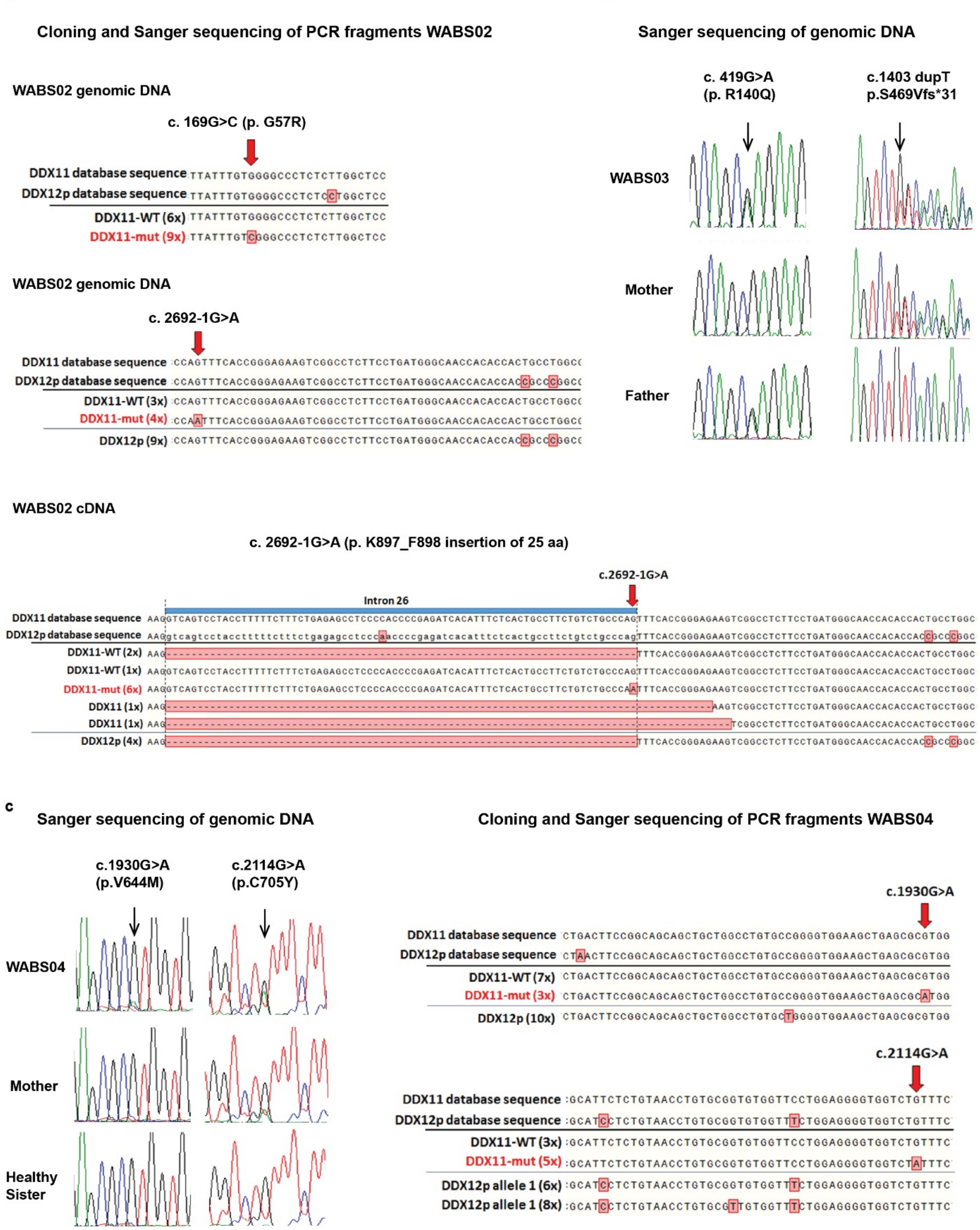

2

d

Sanger sequencing of genomic DNA

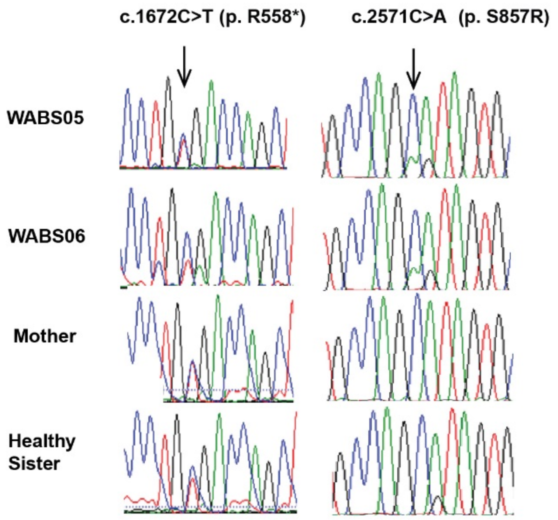

Cloning and Sanger sequencing of PCR fragments WABS05

3

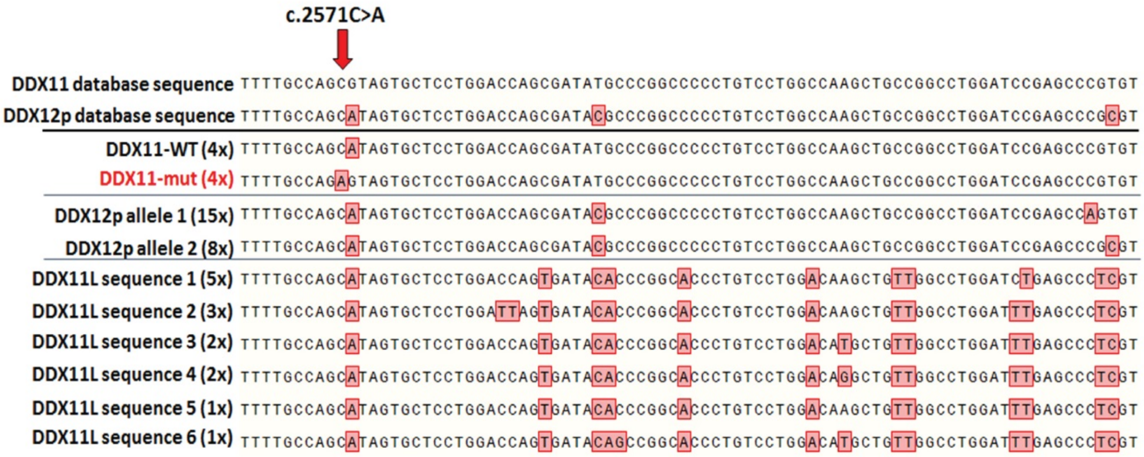

e

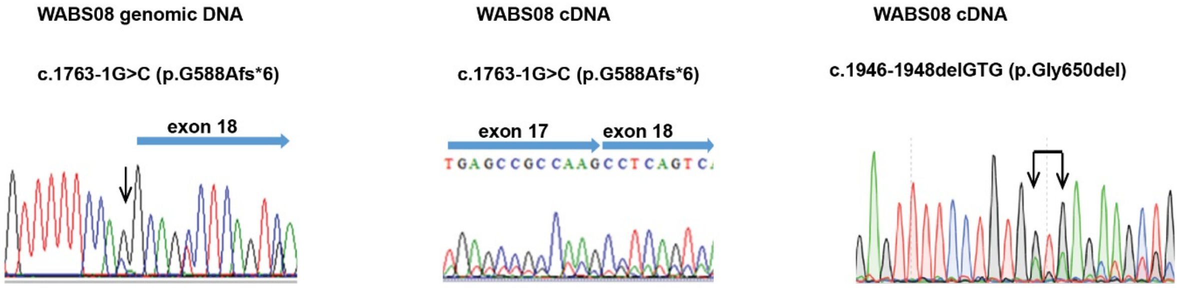

Supplementary Figure 1: Confirmation of pathogenic DDX11 mutations in WABS patients. a) WABS02. PCR fragments, amplified from genomic DNA, were ligated into zero-blunt plasmid, transformed into competent bacteria and DNA from single colonies was sequenced. Sequences were aligned to database DDX11 and DDX12p. One allele contains a c.169G>C missense mutation, leading to a p.G57R substitution. The other allele contains a point mutation affecting the last base of intron 26 (c.2692-1G>A), resulting in retention of intron 26 and in-frame insertion of 25 amino acids at the C-terminus. This was confirmed by sequencing WABS02 cDNA: of seven intron 26 containing reads, six harbored the mutation. b) Sanger sequencing of genomic DNA of WABS03 and both parents. The paternal allele contains a c.419G>A missense mutation, leading to a p.R140Q substitution. The maternal allele contains a c.1403 duplication, leading to a frameshift (p.L468fs*31). c) Sanger sequencing of genomic DNA of WABS04, the mother and a healthy sister. The maternal allele contains a c.2114G>A missense mutation, leading to a p.C705Y substitution. The second allele, not found in the mother and healthy sister, contains a c.1930G>A missense mutation, leading to a p.V644M substitution. To confirm that the mutations are located in DDX11, PCR fragments were ligated into a plasmid and DNA from single colonies was sequenced. Sequences were aligned to database DDX11 and DDX12p. d) Sanger sequencing of genomic DNA of WABS05 and WABS06, the mother and a healthy sister. The maternal allele contains a c.1672C>T nonsense mutation, leading to a premature stop (p.R558*). The second allele contains a c.2571C>A missense mutation, leading to p.S857R substitution. To confirm that mutation c.2571C>A is located in DDX11, PCR fragments were ligated into a plasmid and DNA from single colonies was sequenced. Sequences were aligned to database DDX11 and DDX12p. e, Sanger sequencing of genomic DNA and cDNA of patient WABS08. One allele contains an intronic mutation (c.1763-1G>C) which causes alternative splicing, resulting in deletion of four
4 basepairs in the mRNA and a frameshift (p.G588Afs*6). The second allele contains a splice site mutation, leading to a 3bp deletion in the mRNA (c.1946-1948delGTG) and loss of one amino acid (p.G650del).

Supplementary Figure 2

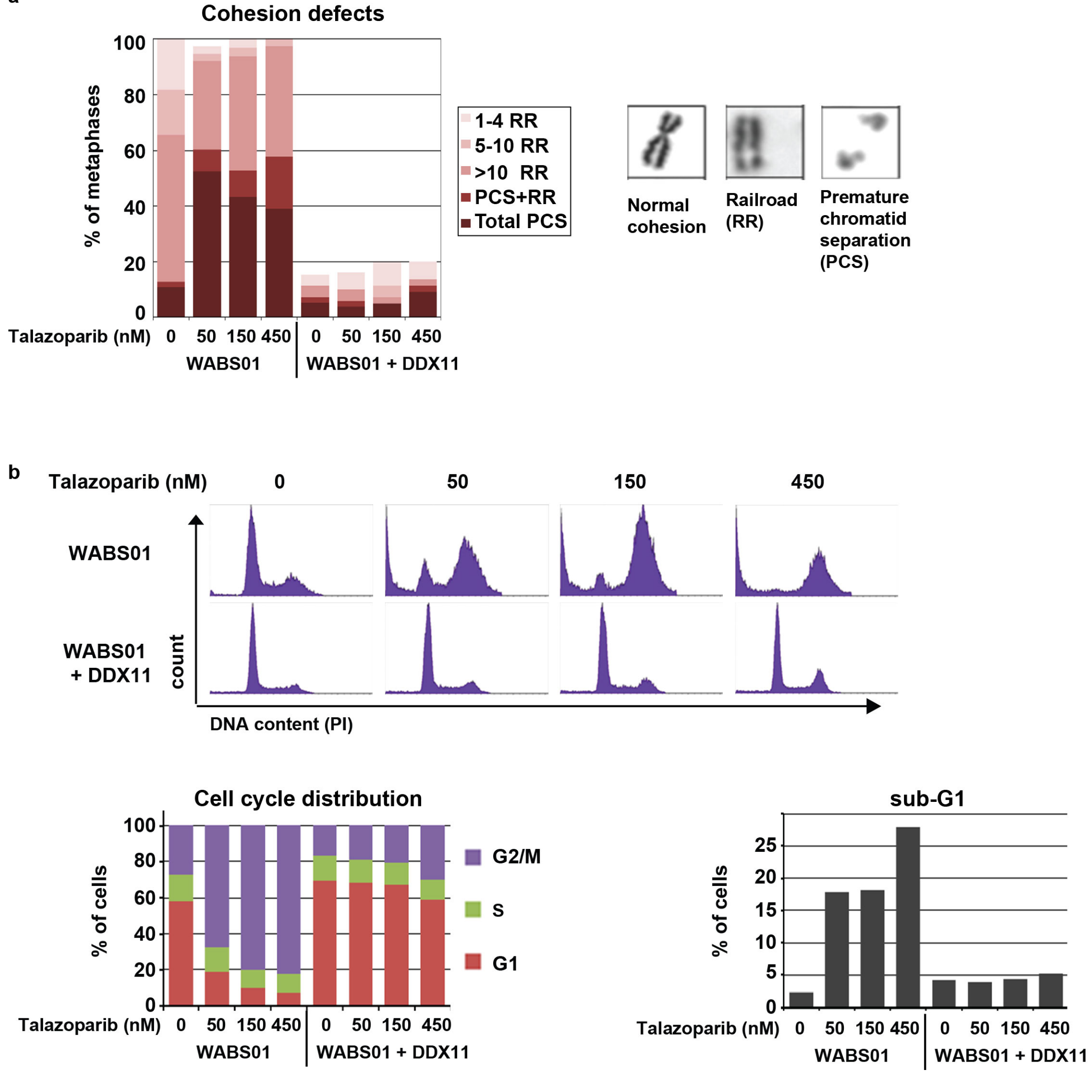

Supplementary Figure 2: Enhanced cohesion loss, G2/M arrest and cell death upon PARPi in WABS fibroblasts. a) cells were treated with indicated doses of talazoparib for 48h and assessed for cohesion defects in 50 metaphases per condition. b) cells were treated with indicated doses of talazoparib for 48h, stained with propidium iodide and analyzed by flow cytometry. Quantifications are shown in the lower graphs.

Supplementary Figure 3

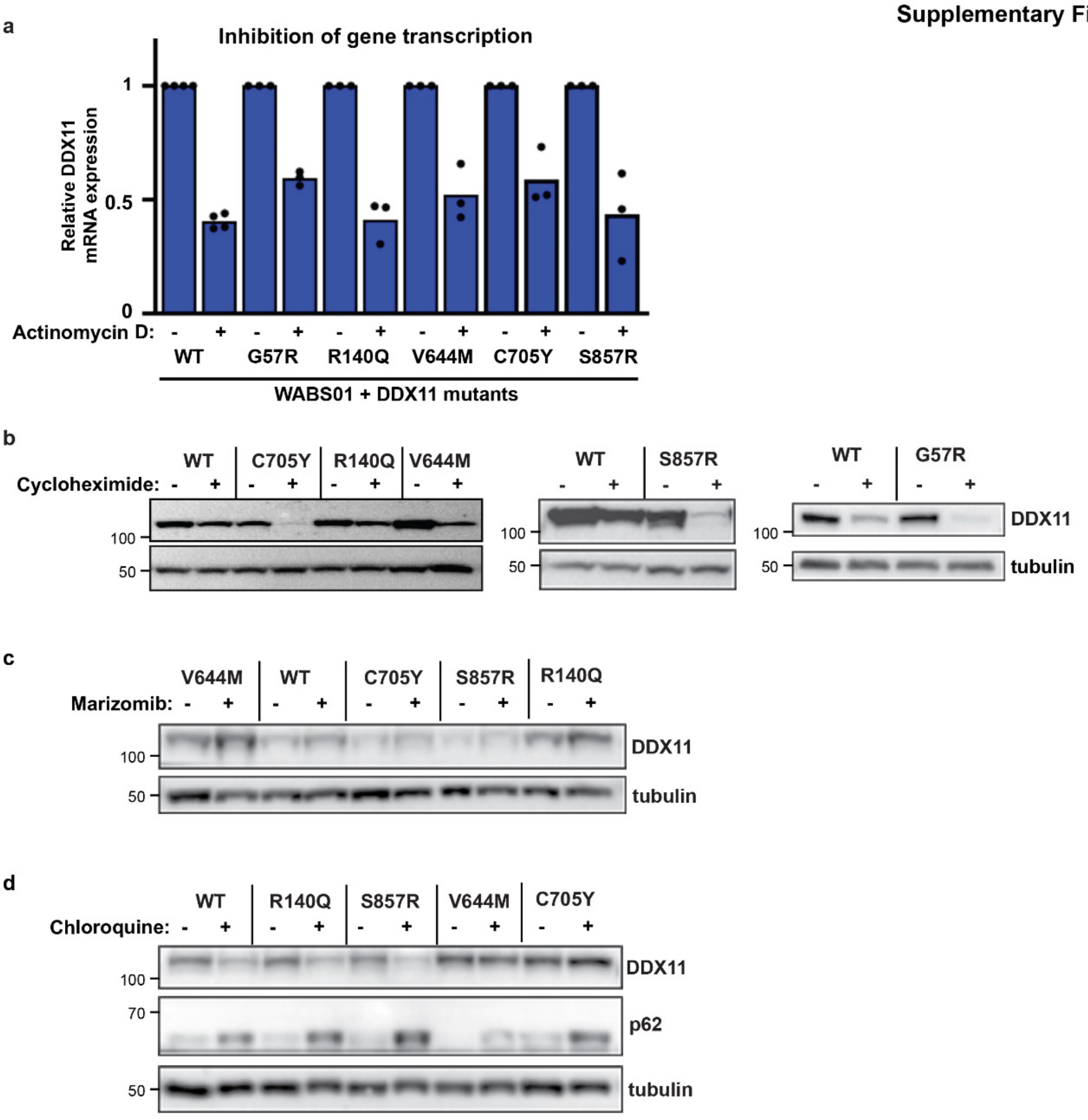

6

Supplementary Figure 3: DDX11 missense alleles reduce protein stability. a) WABS01 cells, stably transfected with cDNAs encoding either WT-DDX11 or several patient-derived DDX11 mutants, were treated with the transcription inhibitor actinomycin D (5 µg/mL, 4h) and DDX11 mRNA levels were analyzed by qRT-PCR. b-d) Cells were treated with cycloheximide (62,5 µg/mL, 3h), marizomib (500 nM, 5h) or chloroquine (25 µM, 24h). Quantifications from at least two independent experiments are provided in Figure 3e and 3f.

Supplementary Figure 4

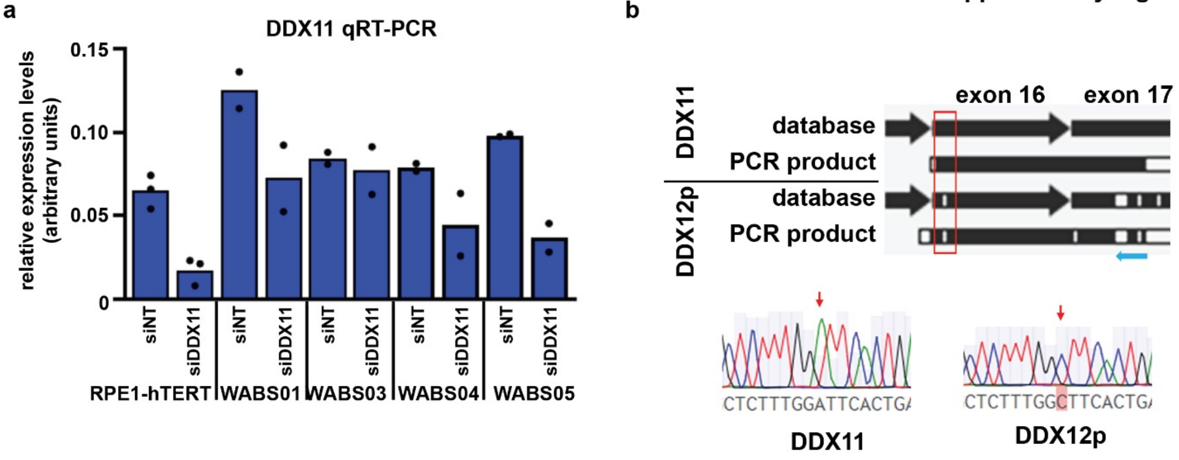

Supplementary Figure 4: Validation of qRT-PCR primers for DDX11 and DDX12p. a) RPE1-hTERT cells and four WABS fibroblasts were transfected with indicated siRNAs (day 1 and day 4) and analyzed for mRNA levels seven days after the first transfection using qRT-PCR. Accompanying cohesion analysis is shown in Figure 3i. b) Specific qRT-PCR primers were designed to distinguish between DDX11 and DDX12p cDNA. This is based on a stretch containing multiple differences in exon 17 (blue arrow). Sanger sequencing of the resulting amplicons confirms specificity of the primers (red arrows).

Supplementary Figure 5

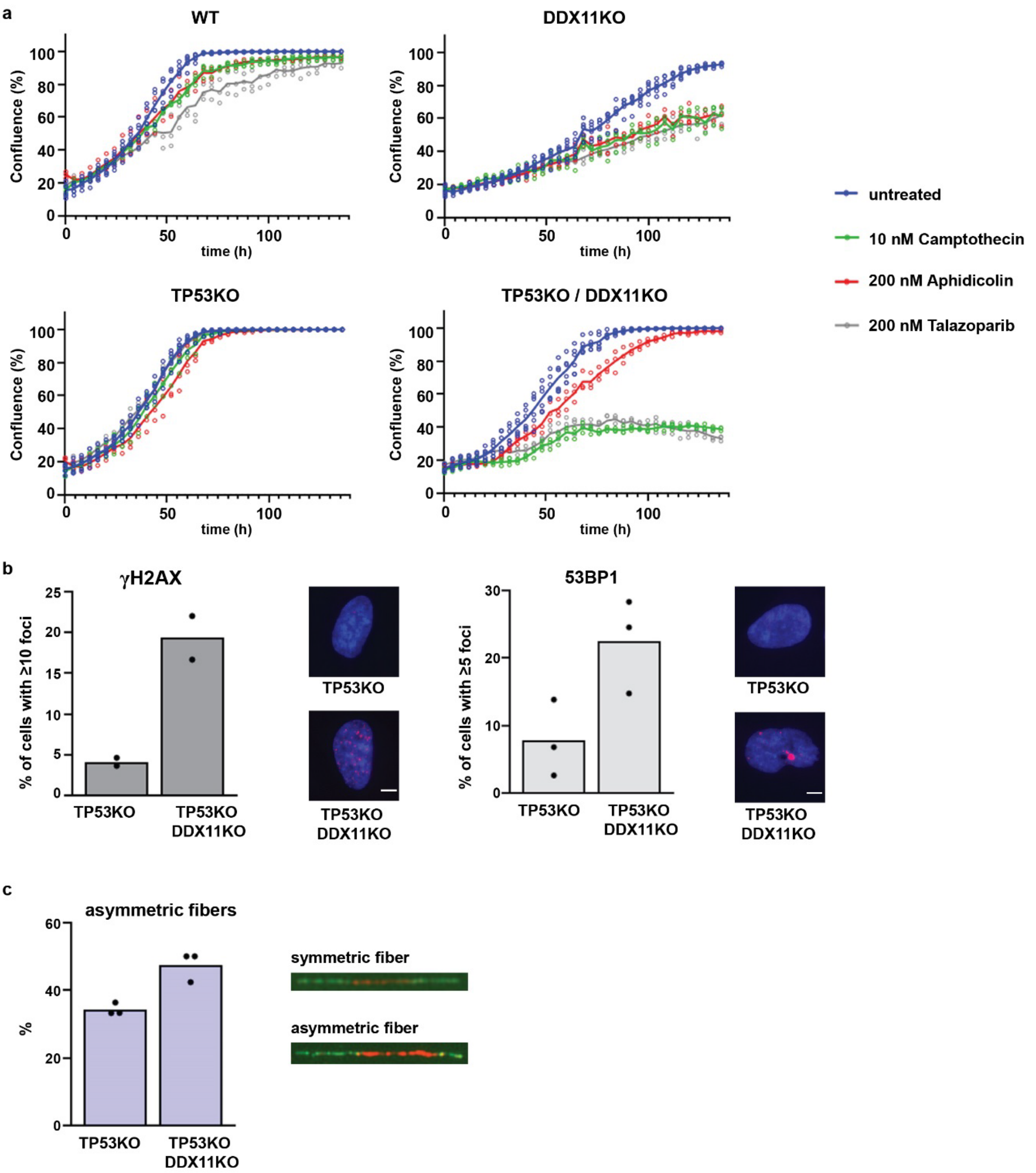

8

Supplementary Figure 5: Drug sensitivity and basal DNA damage signaling in DDX11 deficient cells. a) RPE1 cells were treated with 10 nM camptothecin, 200 nM aphidicolin or 200 nM talazoparib and growth was monitored using IncuCyte software. Three technical replicates are shown. b) Immunofluorescence detection of γH2AX foci (left) and 53BP1 foci (right) of in total 150 cells per condition in three independent experiments. Scale bar is 5 µm. c) A DNA fiber assay was performed using a double labelling protocol and the percentage of asymmetric forks started from first-label origins as percentage of total first-label-origins is indicated. At least 63 first-label origins were scored in three independent experiments. Representative images of symmetric and asymmetric DNA fibers are shown.

9

Supplementary Figure 6

a

clone	DDX11		DDX12p	
	allele 1	allele 2	allele 1	allele 2
wt-TP53_DDX11/12pKO #13	-13	-13	-8	-9
TP53KO_DDX11/12pKO #6	-11	-13	-10	-11
TP53KO_DDX11/12pKO #7	-2	+1	-8	+5
TP53KO_DDX11KO #1B	+226	?	WT	WT
TP53KO_DDX11KO #8A	-258	?	WT	WT
TP53KO_DDX12pKO #C9	WT	+1	+1	+1
TP53KO_DDX12pKO #C14	WT	+1	+1	+1

b

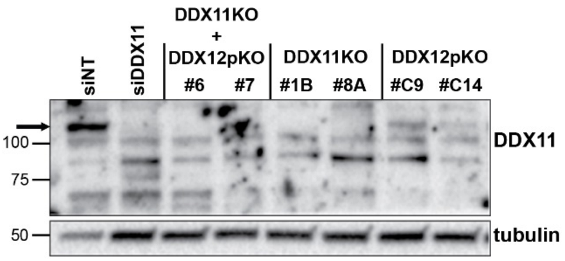

c

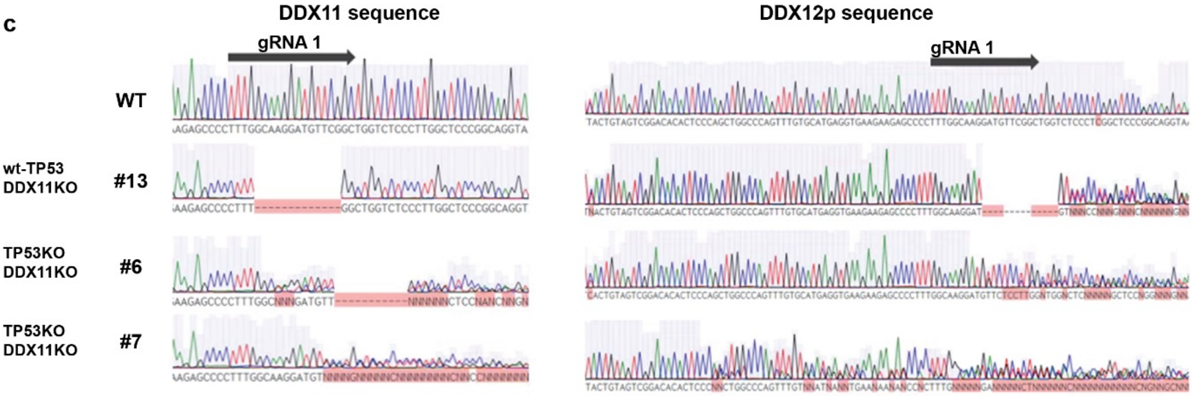

d

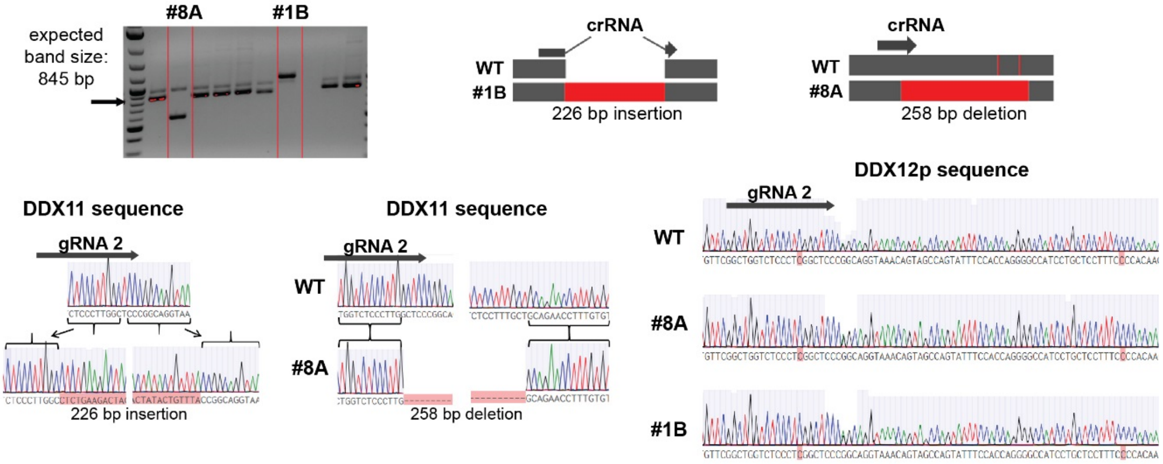

e

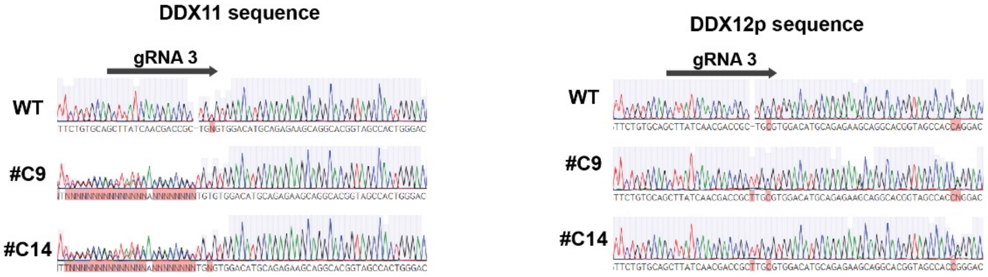

Supplementary Figure 6: Characterization of CRISPR-induced indels in DDX11 and DDX12p. a) Summary of indels present in DDX11 and DDX12p knockout clones. RPE1-hTERT_TetOn-Cas9 cells were treated with 100 ng/mL doxycycline to induce Cas9 expression, transfected with synthetic crRNA and tracrRNA and seeded in limiting dilution. DNA from single clones was PCR amplified using DDX11 and DDX12p specific primers, sequenced and analyzed by TIDE [1] for bi-allelic frameshift mutations. Clone #13 (TP53wt, DDX11KO) and clone #6 (TP53KO, DDX11KO) are also used in Figures 4, 6 and 8. b) A panel of RPE1-TP53KO cells with indicated knockout of DDX11 and/or DDX12p was analyzed for DDX11 and tubulin protein levels by western blot. A representative of tree independent protein analyses is shown. c) Sanger sequencing of indels in DDX11 and DDX12p, created with gRNA 1, confirms frameshifts in the selected clones. d) Analysis of the indels that were created with gRNA2. As we had previously observed that gRNA 2 preferentially causes in-frame indels, we co-transfected a PCR-amplified Zeocin resistance expression cassette, to facilitate gene disruption by Zeocin selection. PCR products from the resulting clones were first analyzed on an agarose gel (performed once), indicating loss of the wild-type band in clones #1B and #8A. Sanger sequencing and alignment with control sequence revealed a large insertion and deletion, respectively. We are uncertain what happened to the other allele in both clones, but considering the absence of wildtype product, DDX11 knockout was confirmed. DDX12p sequences are still intact. e) Sanger sequencing of indels in DDX11 and DDX12p, created with gRNA 3. Note that gRNA 3 not only targets both DDX12p alleles, but also one DDX11 allele in RPE1 cells.

10

Supplementary Figure 7

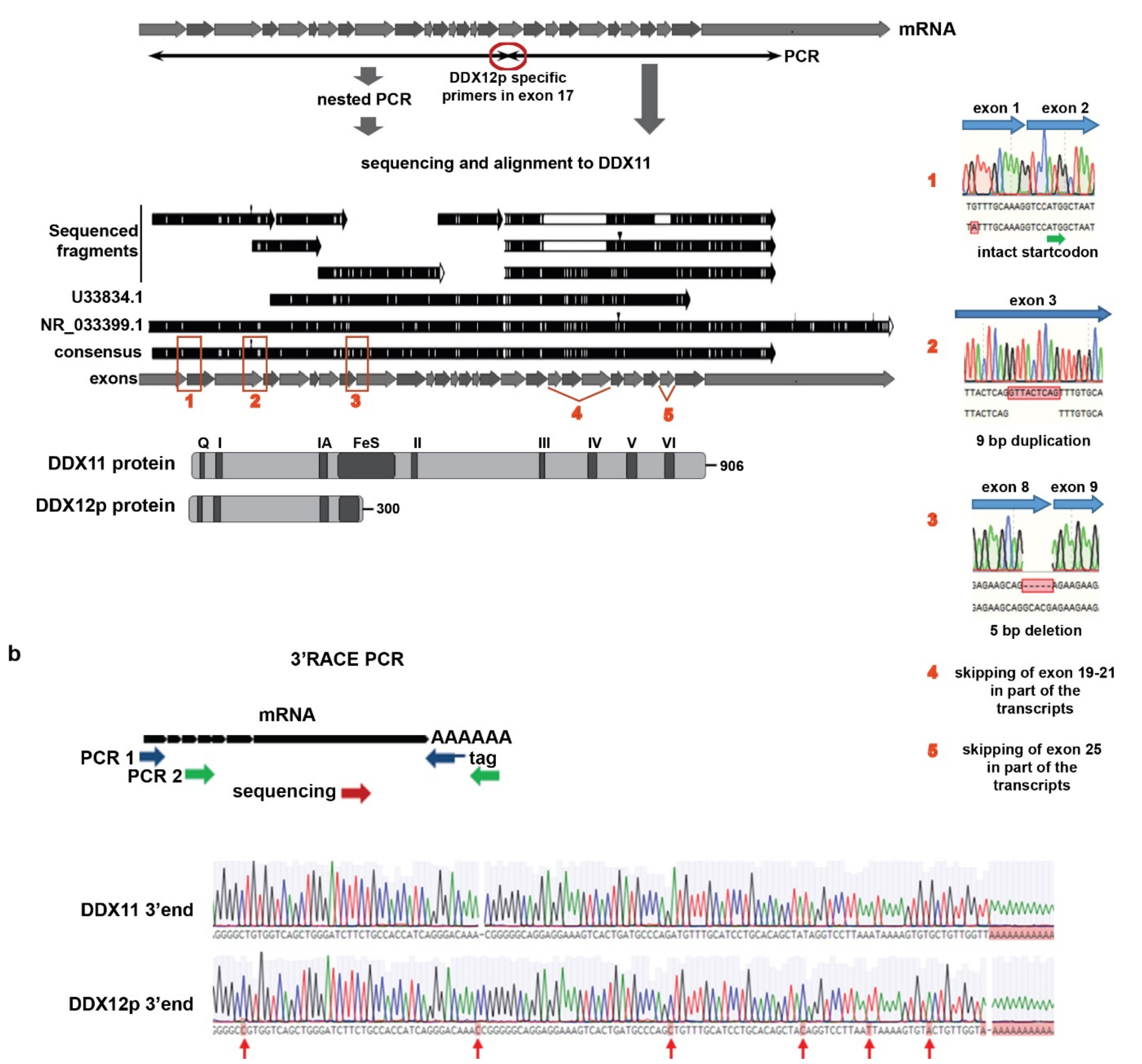

Supplementary Figure 7: Identification of the DDX12p mRNA sequences in RPE1 cells. a) DDX12p cDNA was amplified using the DDX12p-specific reverse primers in exon 17 (Supplementary Figure 4). The exon 1-17 amplicon served as template for several subsequent PCR reactions, using primers that do not discriminate between DDX11 and DDX12p. Both the initial exon 17-27 PCR reaction and the nested PCR fragments were ligated into a zero-blunt plasmid, transformed into competent bacteria and DNA of single colonies was sequenced. Sequences were aligned to DDX11 mRNA as well as to the available database mRNA sequence for DDX12p (NR_033399.1 and U3384.1). We find an intact start codon (1) and a 9bp duplication in exon 3 (2). Importantly, the splicing from exon 8 to exon 9 results in a 5bp deletion in the RPE1 DDX12p transcript as compared with database DDX11 and DDX12p sequences (3), resulting in a frameshift and a truncated protein of 300 amino acids. At the 3’end, we find different isoforms that skip exon 19-21 (4) and/or exon 25 (5), but also one that includes all exons. Consensus DDX12p sequence has been deposited to GenBank, and will be available with accession code MT747418. b) 3’RACE PCR confirms a poly-A tail in DDX12p transcripts. RPE1 RNA was reverse transcribed into cDNA using a DDX11/12p forward primer in exon 27 and an oligo-dT reverse primer. A tag on the reverse prime allows a specific 2nd PCR step, and the resulting PCR fragments were ligated into a zero-blunt plasmid, transformed into bacteria and DNA of single colonies was sequenced. Sequences containing multiple base pairs specifically annotated to DDX12p (database sequence, indicated with red arrows) also contain a poly-A tail.

12

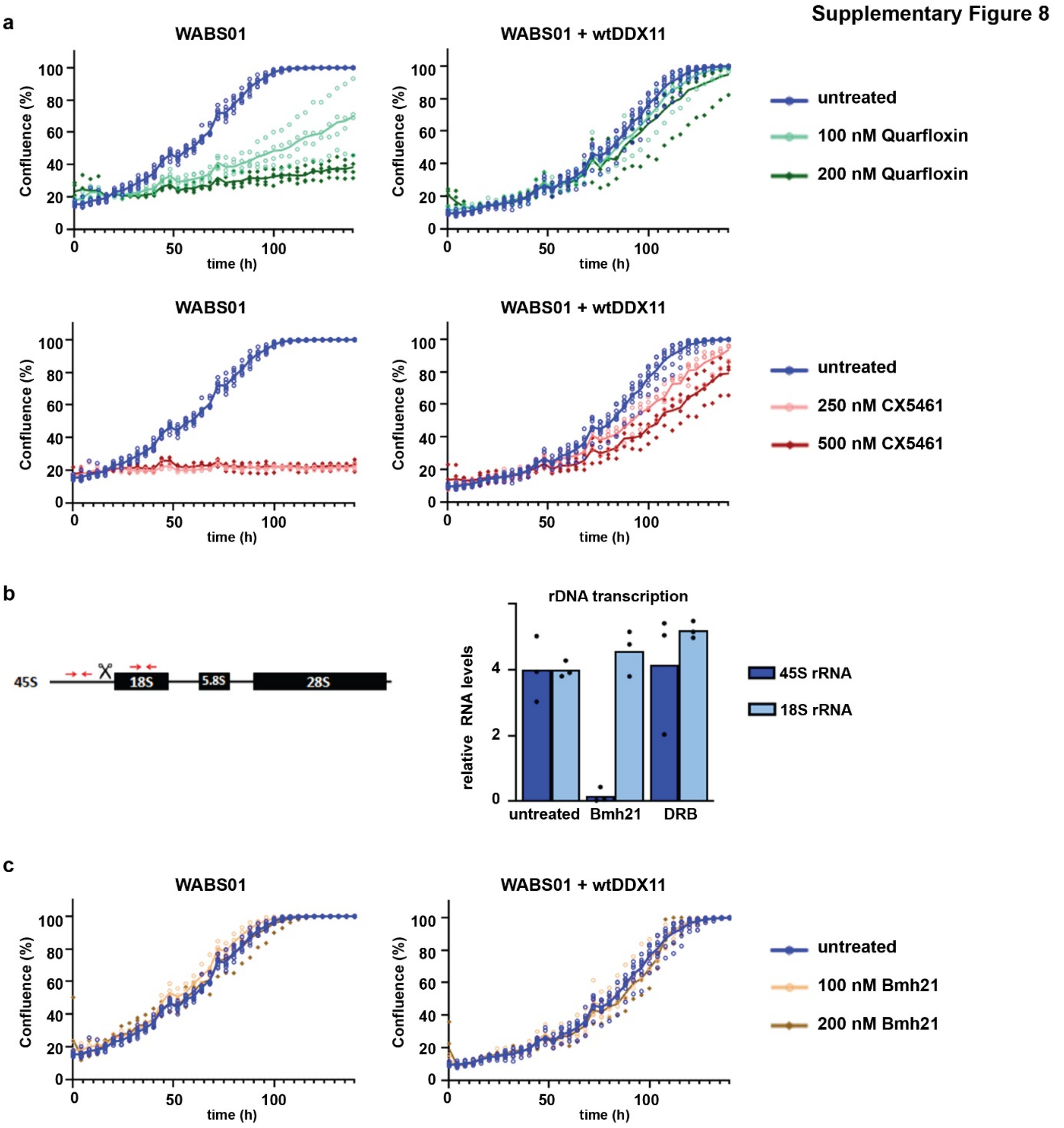

13

Supplementary Figure 8: DDX11 deficiency sensitizes to G4 stabilization but not to inhibition of rDNA synthesis. a) WABS01 and WABS01 + wtDDX11 fibroblasts were cultured in 96-wells plates and treated with quarfloxin or CX5461. Growth was monitored using IncuCyte software in at least four technical replicates. b) RPE1-hTERT-TP53KO cells were treated with the RNA polymerase II inhibitor DRB or the RNA polymerase I inhibitor Bmh21 (1 µM, 7h) and analyzed by qRT-PCR for ribosomal RNA expression. Red arrows indicate primer locations: the 45S rRNA primer pair amplifies a region that is rapidly cleaved off the large precursor transcript, thereby measuring active rDNA transcription rate. The 18S rRNA primer pair amplifies a highly stable region that is present in mature rRNA, thereby serving as control. c) WABS01 and WABS01 + wtDDX11 fibroblasts were cultured in 96-wells plates and treated with Bmh21. Growth was monitored using IncuCyte software in at least four technical replicates.

Supplementary Figure 9

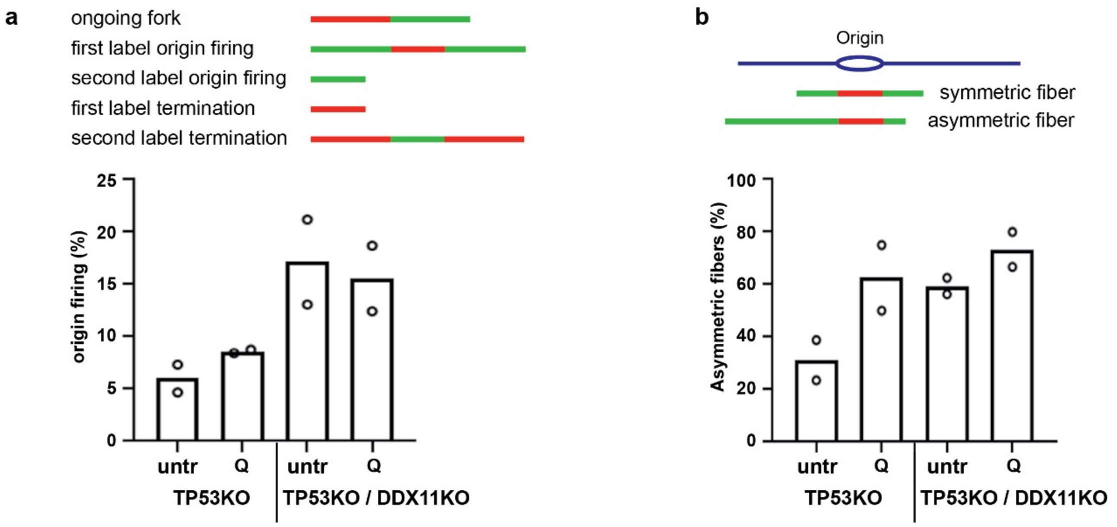

Supplementary Figure 9: Replication fork dynamics upon G4 stabilization and DDX11 depletion. Cells were treated with 200 nM quarfloxin for 24h and assessed with a DNA fiber assay using a double labeling protocol. a) Percentage origin firing was determined for at least 150 fibers in two independent experiments. b) Percentage asymmetric fibers was determined for at least 16 first-label origins in two independent experiments.

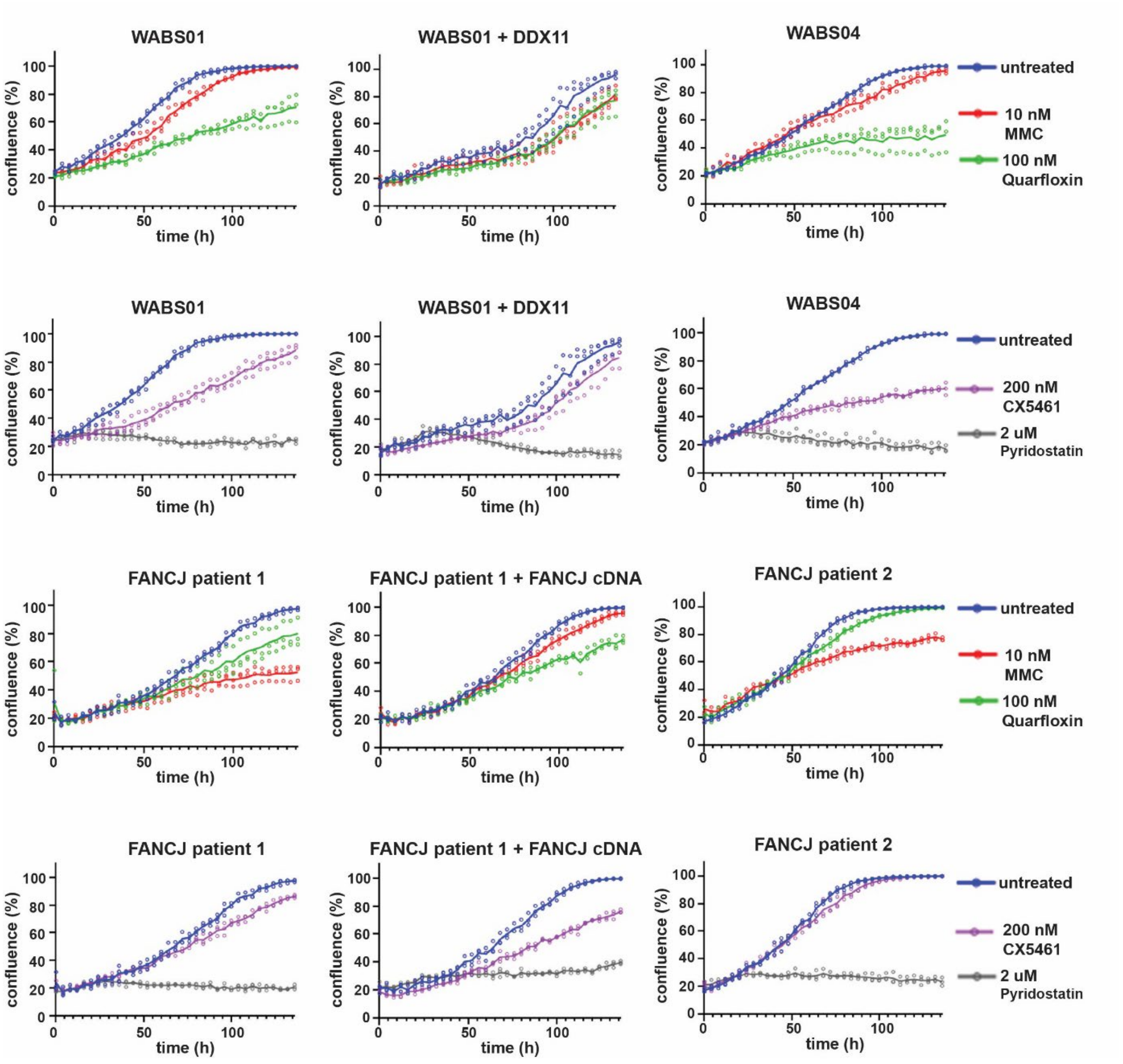

Supplementary Figure 10: Drug sensitivities in WABS cells and FANCJ patient cells. Fibroblasts derived from WABS patients and FANCJ patients were cultured in 96-wells plates and continuously treated as indicated. Growth was monitored using IncuCyte software in three technical replicates.

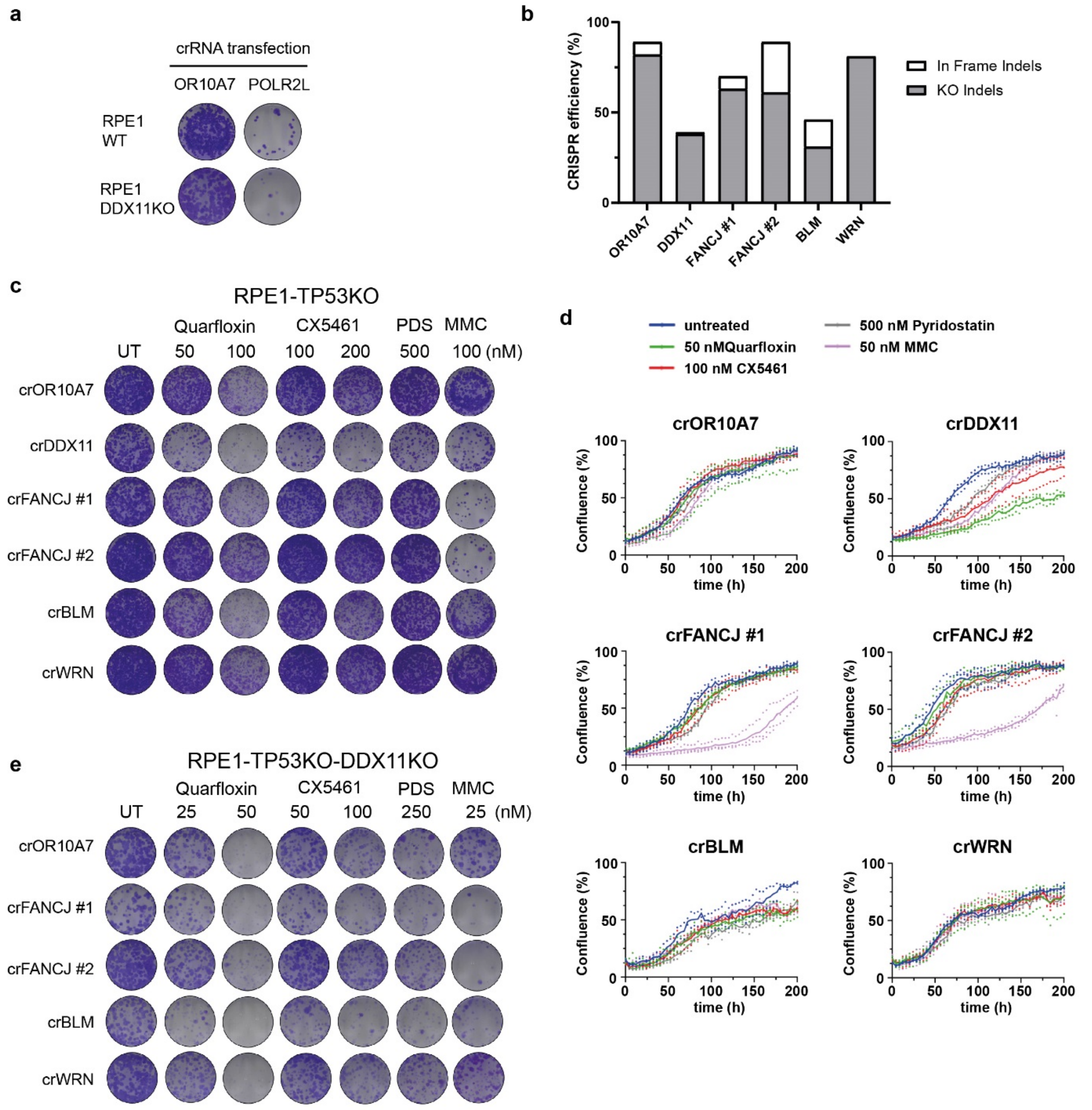

Supplementary Figure 11: Acute gene knockout to assess the role of G4 helicases in G4 stabilizer resistance. a) Clonogenic survival assay was performed of TP53KO and TP53KO-DDX11KO cells two days after Cas9 induction and transfection of synthetic crRNA targeting OR10A7 (negative control) or POLR2L (positive control). b) CRISPR efficiency control by inference of CRISPR edits (ICE) analysis [2]. Percentages of in frame indels and deleterious indels (leading to frameshifts or > 26 bp) at the indicated crRNA target sites are the plotted. c) Clonogenic survival assay of RPE1-TP53KO cells, seeded two days after induction of Cas9 and transfection with indicated crRNAs, treated for 24h as indicated and cultured for another nine days. d) Transfected cells from B were also seeded in 96-wells plates, continuously treated as indicated and growth was monitored using IncuCyte software in three technical replicates. e) RPE1-TP53KO-DDX11KO cells were treated similarly as in b. For all clonogenic survival assays, representative images from two separate experiments are shown.

16

Supplementary Figure 12

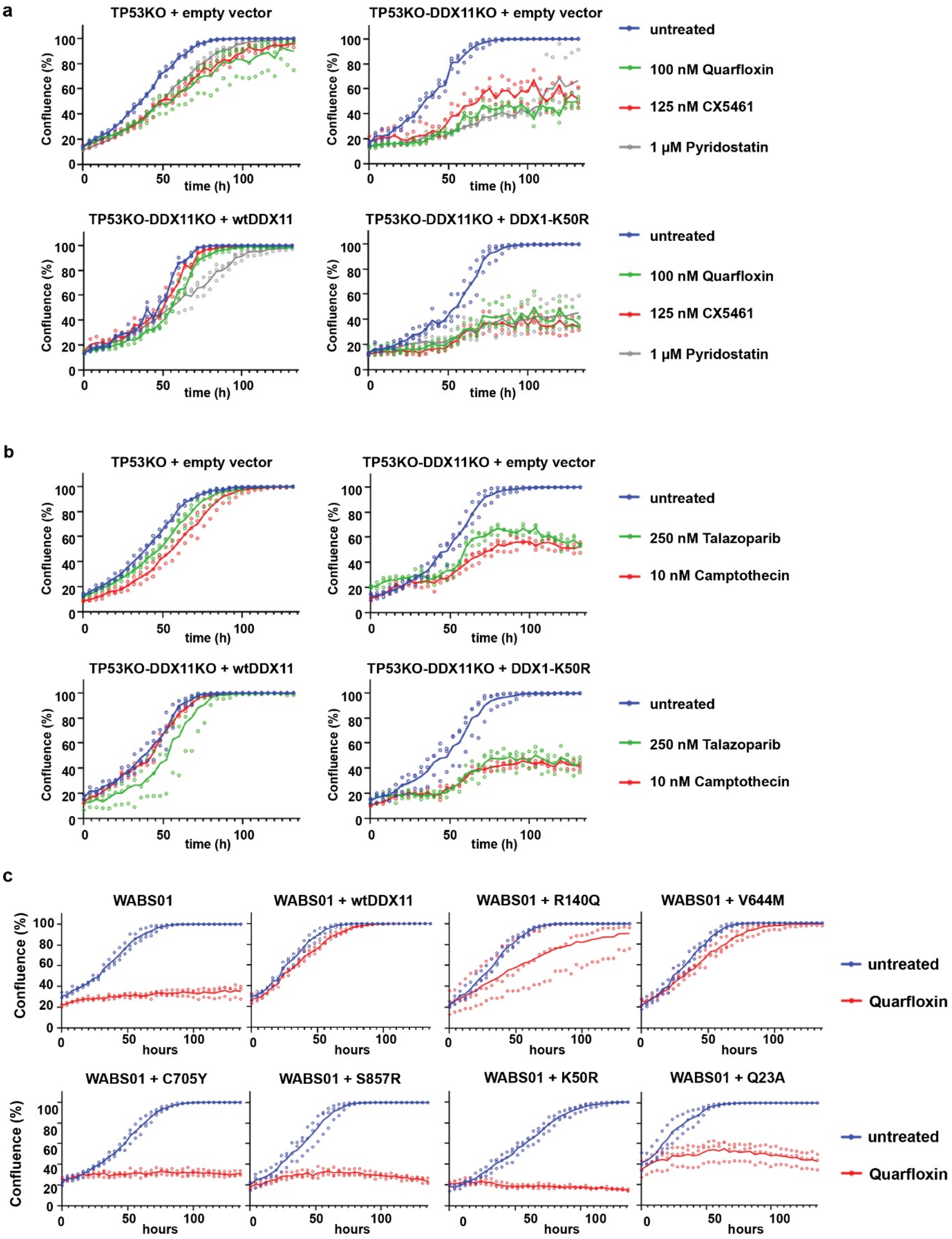

17

Supplementary Figure 12: DDX11 helicase activity is required for resistance to multiple drugs. a) Cells were cultured in 96-wells plates and treated with quarfloxin, CX5461 or pyridostatin. Growth was monitored using IncuCyte software in three technical replicates. b) Similarly, cells were treated with talazoparib or camptothecin and growth was monitored using IncuCyte software. c) Cells were cultured in 96-wells plates and treated with 200 nM quarfloxin. Growth was monitored using IncuCyte software in three technical replicates.

18

Figure 1b

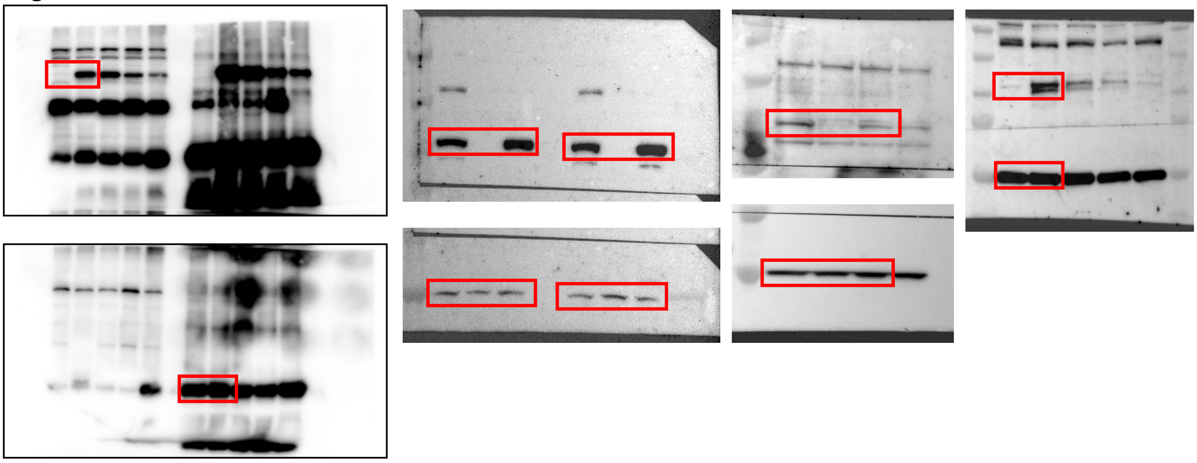

Figure 3a

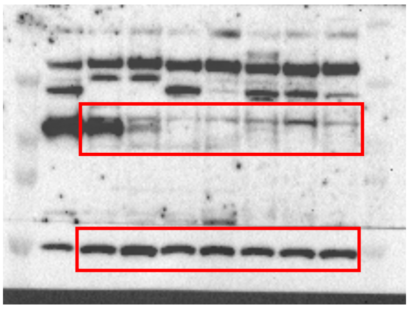

Figure 3c

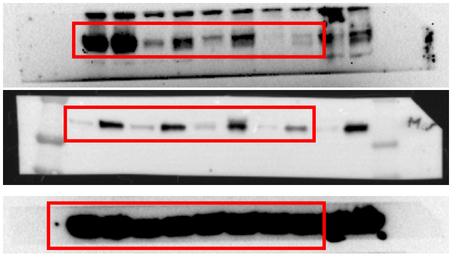

19

Figure 3d

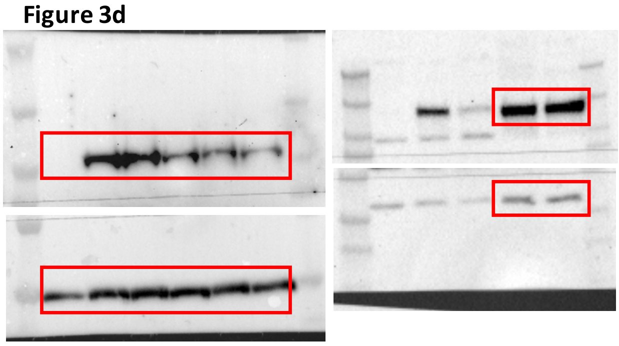

Figure 8a

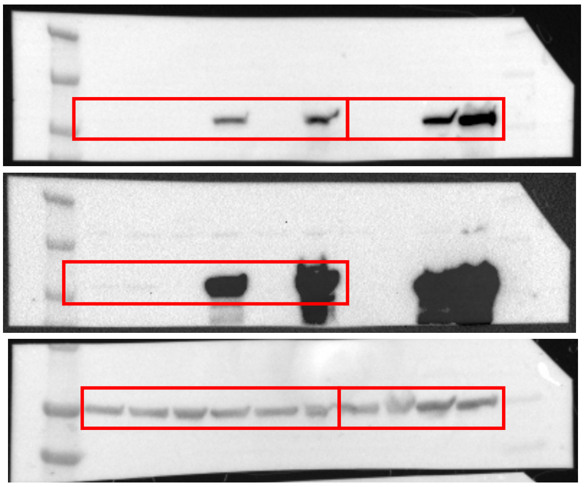

Figure 4b

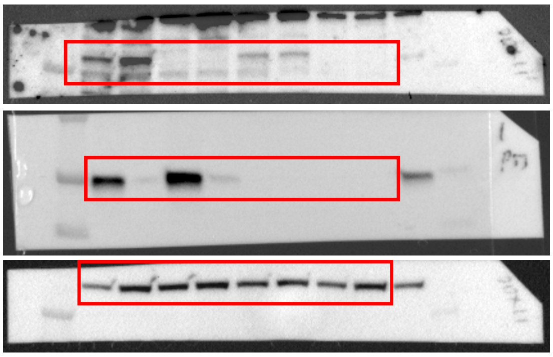

Supplementary Figure 13: uncropped western blots

Supplementary Table 1: Primer sequences

Primer name	Sequence
**3'RACE 1st PCR Fw**	CCAGACAACATCCTGCCCCT
**3'RACE 1st PCR Rv #1**	TGCCCAGTTCCCAGTTCCGATTTTTTTTTTTTTTTTG
**3'RACE 1st PCR Rv #2**	TGCCCAGTTCCCAGTTCCGATTTTTTTTTTTTTTTTC
**3'RACE 1st PCR Rv #3**	TGCCCAGTTCCCAGTTCCGATTTTTTTTTTTTTTTTA
**3'RACE 2nd PCR DDX11 Fw**	ATTCCAGGTGCATCCAGGCC
**3'RACE 2nd PCR DDX12p Fw**	TATTCCAGGTGCGGGCGTCA
**3'RACE 2nd PCR Rv**	TGCCCAGTTCCCAGTTCCGA
**3'RACE DDX11 sequencing primer**	GGCCCTGCTCCCTATCCTGT
**3'RACE DDX12p sequencing primer**	GGCCCTGCTCTCTATCCTGC
**CRISPR indel sequencing TP53 Fw**	GAGACCTGTGGGAAGCGAAA
**CRISPR indel sequencing TP53 Rv**	GCTGCCCTGGTAGGTTTTCT
**CRISPR indel sequencing DDX11 Fw**	AACAACCCACCCTCCCCAAG
**CRISPR indel sequencing DDX11 Rv**	TGCCTCACTCTCTCCAGACC
**CRISPR indel sequencing DDX12p Fw**	GCTGGGTGGT GCTGGATATG
**CRISPR indel sequencing DDX12p Rv**	CTGCCTACTGTGGTCTCATCGG
**CRISPR indel sequencing OR10A7 Fw**	GGATGGCCATAGGCTCTTGGAT
**CRISPR indel sequencing OR10A7 Rv**	CCTCTTGACAGCCCCTTTCACT
**CRISPR indel sequencing FANCJ#1 Fw**	GCCACTGTTCTAGGTGCTGTTG
**CRISPR indel sequencing FANCJ#1 Rv**	CACACCAGAACATAGGCTCCCA
**CRISPR indel sequencing FANCJ#2 Fw**	GCCTGTGGTACCTTGATCCAGA
**CRISPR indel sequencing FANCJ#2 Rv**	AGACACATCCTTCGGGGCTATT
**CRISPR indel sequencing BLM Fw**	tctcagccccaaacgttctgat
**CRISPR indel sequencing BLM Rv**	ctggtcacaaactcccgacctc
**CRISPR indel sequencing WRN Fw**	GTGGGATTTGACATGGAGTGGC
**CRISPR indel sequencing WRN Rv**	ccaggcttaatacccgggtgat
**qRT-PCR DDX11 Fw**	AACCTGTTCAAGGTGCAGCGATAC
**qRT-PCR DDX11 Rv**	GAGAAGCTGGTCGCAGGGT
**qRT-PCR DDX12p Fw**	AACCTGTTCAAGGTGCAGCGATAC
**qRT-PCR DDX12p Rv**	GGGAAGCTGGTTGCGGGAC
**qRT-PCR p21 Fw**	AGCAGAGGAAGACCATGTGGA
**qRT-PCR p21 Rv**	AATCTGTCATGCTGGTCTGCC
**qRT-PCR 45S pre-rRNA Fw**	GCCTTCTCTAGCGATCTGAGAG
**qRT-PCR 45S pre-rRNA Rv**	CCATAACGGAGGCAGAGACA
**qRT-PCR 18S pre-rRNA Fw**	AAACGGCTACCACATCCA
**qRT-PCR 18S pre-rRNA Rv**	CCTCCAATGGATCCTCGT
**qRT-PCR HPRT Fw**	TGACACTGGGAAAACAATGCA
**qRT-PCR HPRT Rv**	GGTCCTTTTCACCAGCAAGCT
**qRT-PCR TBP Fw**	TGCACAGGAGCCAAGAGTGAA
**qRT-PCR TBP Rv**	CACATCACAGCTCCCCACCA
**qRT-PCR B2M Fw**	ATGAGTATGCCTGCCGTGTGA
**qRT-PCR B2M Rv**	GGCATCTTCAAACCTCCATG
**Mouse DDX11 G57R**	CTTAAGTCTGATTTGAC
**Mouse DDX11 WT**	CTTAAGTCTGATTTGTG

20
