# Supplementary material for: Warsaw Breakage Syndrome associated DDX11 helicase resolves G-quadruplex structures to support sister chromatid cohesion
Source: Nat Commun. 2020 Aug 27;11:4287. doi: 10.1038/s41467-020-18066-8 (PMC7452896; doi:10.1038/s41467-020-18066-8)
Supplement: Supplementary file 3 — Reporting Summary [file 41467_2020_18066_MOESM3_ESM.pdf]

nature research

Corresponding author(s): Rob Wolthuis and Job de Lange
Last updated by author(s): Jul 20, 2020

Reporting Summary

Nature Research wishes to improve the reproducibility of the work that we publish. This form provides structure for consistency and transparency in reporting. For further information on Nature Research policies, see our Editorial Policies and the Editorial Policy Checklist.

Statistics

For all statistical analyses, confirm that the following items are present in the figure legend, table legend, main text, or Methods section.

n/a	Confirmed	
☐	☒	The exact sample size (n) for each experimental group/condition, given as a discrete number and unit of measurement
☒	☐	A statement on whether measurements were taken from distinct samples or whether the same sample was measured repeatedly
☐	☒	The statistical test(s) used AND whether they are one- or two-sided<br>*Only common tests should be described solely by name; describe more complex techniques in the Methods section.*
☒	☐	A description of all covariates tested
☒	☐	A description of any assumptions or corrections, such as tests of normality and adjustment for multiple comparisons
☐	☒	A full description of the statistical parameters including central tendency (e.g. means) or other basic estimates (e.g. regression coefficient) AND variation (e.g. standard deviation) or associated estimates of uncertainty (e.g. confidence intervals)
☐	☒	For null hypothesis testing, the test statistic (e.g. F, t, r) with confidence intervals, effect sizes, degrees of freedom and P value noted<br>*Give P values as exact values whenever suitable.*
☒	☐	For Bayesian analysis, information on the choice of priors and Markov chain Monte Carlo settings
☒	☐	For hierarchical and complex designs, identification of the appropriate level for tests and full reporting of outcomes
☒	☐	Estimates of effect sizes (e.g. Cohen's d, Pearson's r), indicating how they were calculated

Our web collection on statistics for biologists contains articles on many of the points above.

Software and code

Policy information about availability of computer code

Data collection	No software was used to collect data
Data analysis	Western blots and DNA gels were analyzed and (where applicable) bands were quantified using Image Lab version 3.0 software (BioRad)<br>qRT-PCR experiments were analyzed using LightCycler 480 (Roche)<br>Metaphase spreads were assessed with Metafer 4 v3.11.3<br>Fluorescent microscopy slides (both cells and DNA fiber assays) were analyzed using Leica microscope and ImageJ v1.8.0 software<br>Image preparations were performed with Photoshop CS6<br>Graph design and statistical analysis was done with GraphPad Prism 8.2.1<br>Incucyte experiments were analysed wtih IncuCyte ZOOM 2018A software<br>SureCall software version 2.0 was used to detect genetic variants<br>Cell cycle analysis was performed with BD CellQuest Pro v6.0 software<br>For indel formation analysis after CRISPR-Cas9 Synthego inference of CRISPR edits (ICE) v2.0 was used<br>Exome data analysis was performed using genome analysis tool kit (GATK4; www.broadinstitute.org/gatk).

For manuscripts utilizing custom algorithms or software that are central to the research but not yet described in published literature, software must be made available to editors and reviewers. We strongly encourage code deposition in a community repository (e.g. GitHub). See the Nature Research guidelines for submitting code & software for further information.

April 2020

Data

Policy information about availability of data

All manuscripts must include a data availability statement. This statement should provide the following information, where applicable:

- Accession codes, unique identifiers, or web links for publicly available datasets
- A list of figures that have associated raw data
- A description of any restrictions on data availability

DDX11 sequencing reads from WABS patients have been deposited to the Sequence Reads Archive (SRA) with accession code PRJNA645773. For sequence alignments, we used Homo sapiens chromosome 12, GRCh38.p13 Primary Assembly (accession NC_000012.12, which contains both DDX11 and DDX2p gene sequences); as well as DDX11 transcript, accession NM_030653.4, and DDX12p transcript, accession NR_033399.1. The here obtained sequence of the DDX12p transcript of RPE1-TERT cells has been deposited to GenBank, and will be available with accession code MT747418. All other relevant data supporting the key findings of this study are available within the article and its Supplementary Information files, the source data, or from the corresponding authors upon reasonable request.

Field-specific reporting

Please select the one below that is the best fit for your research. If you are not sure, read the appropriate sections before making your selection.

☒ Life sciences ☐ Behavioural & social sciences ☐ Ecological, evolutionary & environmental sciences

For a reference copy of the document with all sections, see nature.com/documents/nr-reporting-summary-flat.pdf

Life sciences study design

All studies must disclose on these points even when the disclosure is negative.

Sample size	No statistical methods were used to predetermine sample size. Sample sizes were determined based on published sample size usages for similar experiments, examples include (Xu, H. et al. CX-5461 is a DNA G-quadruplex stabilizer with selective lethality in BRCA1/2 deficient tumours. Nat Commun 8, 14432 (2017); Benedict, B. et al. WAPL-Dependent Repair of Damaged DNA Replication Forks Underlies Oncogene-Induced Loss of Sister Chromatid Cohesion. Dev Cell 52, 683-698 e7 (2020); Leman, A.R., Noguchi, C., Lee, C.Y. & Noguchi, E. Human Timeless and Tipin stabilize replication forks and facilitate sister-chromatid cohesion. J. Cell Sci 123, 660-670 (2010))
Data exclusions	No data were excluded.
Replication	measurements were performed in at least two independent experiments. All results were reproducible.
Randomization	No randomization was required. Samples were treated according to the same protocols side-by-side with the respective controls.
Blinding	For microscopic evaluations, slides were coded prior to analysis to secure blinding. No blinding was performed for the other experiments; all experiments were performed multiple times to ensure reproducibility.

Reporting for specific materials, systems and methods

We require information from authors about some types of materials, experimental systems and methods used in many studies. Here, indicate whether each material, system or method listed is relevant to your study. If you are not sure if a list item applies to your research, read the appropriate section before selecting a response.

Materials & experimental systems

n/a	Involved in the study	
☐	☒	Antibodies
☐	☒	Eukaryotic cell lines
☒	☐	Palaeontology and archaeology
☐	☒	Animals and other organisms
☐	☒	Human research participants
☒	☐	Clinical data
☒	☐	Dual use research of concern

Methods

n/a	Involved in the study	
☒	☐	ChIP-seq
☒	☐	Flow cytometry
☒	☐	MRI-based neuroimaging

Antibodies

Antibodies used	mouse anti-DDX11 (B01P, Abnova), goat anti-β-actin (I-19, Santa Cruz), mouse anti-α-tubulin (B-5-1-2, Santa Cruz #sc-23948), mouse anti-CDC6 (Santa Cruz #sc-9964), mouse anti-p62 (D5L7G, cell signaling), mouse anti-Flag (M2, Sigma), mouse anti-p53 (DO-1, Santa Cruz #sc-126), mouse anti-vinculin (H-10, Santa Cruz #sc-25336), guinea pig anti-ESCO2 (Van der Lelij et al, PLoS One, 2009),

peroxidase-conjugated secondary antibodies (DAKO Glostrup, Denmark)

Validation

Bands of correct molecular weights were detected from anti-β-actin, anti-α-tubulin, anti-CDC6, anti-p62, anti-p53 and anti-vinculin by Western blot analysis of whole cell lysate of human cell lines. For anti-DDX11, anti-Flag and anti-ESCO2, bands of correct molecular weight were detected from cell lines transfected with recombinant protein.

Eukaryotic cell lines

Policy information about cell lines

Cell line source(s)

RPE1-hTERT: retinal pigment epithelial cell line, purchased from the American tissue culture collection. A fibroblast cell line from patient WABS02 was established from a skin biopsy, in the Institute of Cancer and Genomic Sciences, University of Birmingham, UK. From patient WABS03, a fibroblast cell line was established from a skin biopsy and a lymfoblast cell line was established from a blood biopsy, in the Departamento de Genetica, Hospital Militar, Montevideo, Uruguay. From patient WABS05, a fibroblast cell line was established from a skin biopsy and a lymfoblast cell line was established from a blood biopsy, and from patient WAB06 a lymfoblast cell line was established from a blood biopsy, in the Children's Hospital Zagreb, Center of Excellence for Reproductive and Regenerative Medicine, Medical School University of Zagreb, Croatia. A fibroblast cell line from patient WABS08 was established form a skin biopsy, in Department of Clinical Genetics, Erasmus Medical Center, Rotterdam, The Netherlands. From patient WABS04, FANCJ patients and healthy controls, lymphoblast cell lines were established from blood biopsies and fibroblast cell lines were established from skin biopsies in the department of Clinical Genetics, Amsterdam UMC, Amsterdam, Netherlands. RPE1-hTERT cells and fibroblast cell lines were cultured in Dulbecco's Modified Eagles Medium supplemented with 10% FCS, 1 mM sodium pyruvate and antibiotics. Lymphoblast cell lines were cultured in Roswell Park Memorial Institute supplemented with 10% FCS, 1 mM sodium pyruvate and antibiotics.

Authentication

None of the cell lines used were authenticated

Mycoplasma contamination

All cell lines tested negative for mycoplasma conatamination.

Commonly misidentified lines
(See ICLAC register)

No commonly misidentified cell lines were used in the study.

Animals and other organisms

Policy information about studies involving animals; ARRIVE guidelines recommended for reporting animal research

Laboratory animals

Mutant mice were generated by injecting mutant ES cells into C57Bl/6 blastocysts. Breeding pairs were formed of animals between 2 and 6 months of age; embryos were genotyped for DDX11 allele status, irrespective of sex. Animals were housed in open cages in rooms with an ambient temperature of 21 oC, 55% humidity and a dark/light cycle of 12 hours (07:00-19:00).

Wild animals

The study did not involve wild animals.

Field-collected samples

The study did not involve samples collected from field.

Ethics oversight

All animal study protocols were approved by the NKI Animal Welfare Body.

Note that full information on the approval of the study protocol must also be provided in the manuscript.

Human research participants

Policy information about studies involving human research participants

Population characteristics

We included material derived from Warsaw Breakage Syndrome patients in our studies. These include both male and female patients from different locations.
WABS02 (male) is the second child of Dutch parents, initially diagnosed with Nijmegen Breakage Syndrome (NBS), although no NBS1 mutations were found 44. He showed growth retardation, microcephaly, deafness and abnormal skin pigmentation. WABS03 (male) is the second child of Uruguayan parents. He received pediatric intensive care for several months after birth due to respiratory problems, showed severe developmental delay, microcephaly, sensorineural deafness, hyperactivity and multiple broncho-obstructive episodes. Also congenital hypothyroidism, low set ears and retrognathia were observed. WABS04 (female) is the first child of Dutch parents, initially diagnosed with FA with unknown genetic cause 45. She was born at the 7th month of pregnancy weighing 750 g and had epileptic episodes at the age of three, and childhood hyperactivity as described by the mother. At the age of 45, the following clinical features were recorded: growth and mental retardation, deafness, microcephaly, skin pigmentation (café-au-lait spots), facial dysmorphy, bulbous nose, clinodactyly of the 5th fingers, insulin-dependent diabetes mellitus and frequent respiratory and middle-ear infections. No typical indications of anemia or malignancies were observed. She died at the age of 64, no autopsy report is available. WABS04 had three unaffected siblings and a brother with clinical features that may have been overlapping. The brother was likely affected, too, but he died of heart failure at the age of 50 before a WABS diagnosis was confirmed 45. WABS05 (male) is the fourth child of non-consanguineous parents from Croatia. He showed pre-natal growth retardation; his birth weight, after 36 weeks, was 1660 g. He suffered epileptic seizures at the age of seven and displayed brachy-microcephaly, moderate to severe intellectual disability, bronchial asthma, clinodactyly of the 5th fingers, flexion contractures of thumbs and sandal gap of toes and he is deaf-mute. Cytogenetic investigation showed a 47XXY karyotype and cohesion defects. WABS06 (female) is the older sister of WABS05. Her birth weight, after 37 weeks, was 2100 g. She shows brachy-microcephaly, abnormal skin pigmentation (café-au-lait spots), clinodactyly of the 5th fingers, sandal gap of toe and is deaf-mute. At early age, her intellectual development was estimated to be normal but later declined. WABS07 (male) is a fetus of Dutch parents. The pregnancy was prematurely abrogated due to severe growth restriction of the fetus and placental abnormalities. Furthermore, the fetus showed mild

	dysmorphic characteristics, lung hypoplasia, increased liver-brain ratio, unilateral kidney dysplasia and skin abnormalities. WABS08 (male) is a younger fetus of the same parents, also from a pregnancy prematurely abrogated due to severe embryonic growth retardation.
Recruitment	Patients were referred to us based on clinical and/or cytogenetic signs of Warsaw Breakage Syndrome, to perform detailed investigations of DDX11 mutations and cellular characteristics that are associated with this syndrome.
Ethics oversight	The research on patient material was carried out after approval by the institutional review board of the VU University Medical Center, adhered to local ethical standards. Appropriate informed consent was obtained from patients or from parents/relatives as applicable.

Note that full information on the approval of the study protocol must also be provided in the manuscript.
